# An Automated Diagnosis of Myopia from an Optic Disc Image Using YOLOv11: A Feasible Approach for Non-Expert ECPs in Computer Vision

**DOI:** 10.3390/life15101495

**Published:** 2025-09-23

**Authors:** Nicola Rizzieri, Luca Dall’Asta, Maris Ozoliņš

**Affiliations:** 1 Department of Optometry and Vision Science, The Faculty of Science and Technology, University of Latvia, Jelgavas Street 1, LV-1004 Riga, Latvia; maris.ozolins@lu.lv; 2 LIFE Srl, Research and Development, IT-70100 Bari, Italy; dallas.luca@gmail.com; 3 Institute of Solid State Physics, University of Latvia, Kengaraga Street 8, LV-1063 Riga, Latvia

**Keywords:** myopia, optic disc, fundus photography, YOLO, computer-aided diagnosis, screening tool

## Abstract

Myopia is a common refractive error with a rising prevalence worldwide, and its early diagnosis is crucial to prevent long-term visual impairment. This study presents an accessible, automated approach for detecting myopia from fundus photographs by analyzing the optic disc, using a deep learning model based on the YOLO (You Only Look Once) architecture, version 8 and 11. The pipeline was designed to be usable by eye care practitioners (ECPs) with no expertise in computer science. Fundus images were processed to extract the optic disc region using a custom-trained YOLOv8 model, and a subsequent classification algorithm determined the presence or absence of myopia based on features from the extracted region. The system was trained on a single-clinic dataset of 730 augmented images, with 98 images reserved for internal validation, and tested on 50 independent optic disc images. It achieved a high diagnostic accuracy, with strong sensitivity and F1 scores. Lightweight models such as YOLOv11-nano performed comparably to larger variants in the testing dataset (AUC 97.5% vs. 97.3%), effectively supporting myopia detection. This work highlights the feasibility of integrating AI-based screening tools into clinical practice without requiring advanced technical skills, offering a scalable and cost-effective solution to support early diagnosis of myopia in diverse healthcare settings.

## 1. Introduction

Myopia is a prevalent refractive error in which distant objects appear blurred because light focuses in front of the retina, usually due to axial elongation [1]. It affects about one third of children and adolescents worldwide, with rates approaching 45–47% in some groups, and its prevalence is projected to reach 40% by 2050 [2]. Diagnosis typically relies on visual acuity testing, cycloplegic refraction, and axial length measurements. Recently, artificial intelligence (AI) has been investigated as a tool to develop alternative diagnostic methods, enabling earlier detection [3]. Early myopia diagnosis is recommended to reduce the burden of side-eye threatening conditions [4].

Characteristic fundus changes in myopic eyes include an elliptical, tilted optic disc (quantified by the ovality index), myopic crescent, and parapapillary atrophy (PPA), which enlarges with increasing axial length [5,6,7]. These features correlate with disease severity and provide useful landmarks for automated image analysis based on fundus photography or optical coherence tomography (OCT).

Several studies have investigated the potential of fundus features for the automated diagnosis of myopia. For instance, studies using deep learning on fundus images have demonstrated robust detection of disc tilt patterns, achieving an area under the receiver operating characteristic curve (AUC) of ~0.98 [2]. Similarly, automated segmentation of fundus tessellation, optic disc area, PPA and tessellated density using convolutional neural networks (CNNs) has shown significant correlations with refractive error and axial length in young adults [3]. In the context of high myopia and pathologic myopia, other works have applied CNN models for optic disc and lesion segmentation, such as in the PALM (Pathologic Myopia) challenge [5], reaching an AUC ~0.99 for pathological myopia. Varadarajan et al. developed deep learning models to predict refractive error from fundus photographs, achieving a mean absolute error of less than 1 diopter [6].

The development and validation of AI-driven software for medical applications is a complex and multidisciplinary task that typically demands a solid foundation in programming, data processing, algorithm design, and machine learning (ML) theory. These competencies, however, are not commonly found among healthcare professionals, whose training is usually focused on clinical knowledge and patient care rather than computational methodologies. This knowledge gap can significantly hinder the adoption and development of AI tools in clinical environments. To mitigate these barriers and democratise access to artificial intelligence in medicine, we leveraged a pre-trained online application programming interface (API) incorporating state-of-the-art machine learning capabilities. These APIs abstract away the technical complexities of model architecture, training pipelines, and parameter tuning, allowing users with only basic computer literacy to build, train, and deploy functional predictive models with minimal coding. This approach enables faster prototyping and broader clinical engagement, especially in settings with limited technical support [7].

In a previously published paper related to this work, we investigated the potential of AI to analyze retinal fundus images for the detection of myopia [8]. The study achieved a promising overall accuracy of 84%, with a recall of 97% and an F1 score of 89.5%, indicating excellent sensitivity in detecting myopic cases. The AUC reached 82%, underscoring the robustness of the best-performing model.

These results validate the feasibility of combining optic disc localization with AI-powered classification algorithms to support accurate and scalable myopia screening. By utilizing accessible AI tools such as pre-trained APIs, non-experts can now participate meaningfully in the development of diagnostic software, thus bridging the gap between clinical expertise and technological innovation [9,10]. This shift has the potential to accelerate the integration of machine learning into everyday clinical practice, particularly for resource-limited or underserved areas.

Building on this evidence, the present study introduces an automated and user-friendly diagnostic pipeline, enabling healthcare professionals with no computer science background to (i) develop their own algorithm with data coming from their practice; (ii) extract the optic disc using a custom YOLOv8 model [11]; and (iii) classify the presence of myopia by analyzing the extracted optic disc with its intrinsic characteristics, as described above.

The aim is to create an accessible, scalable AI pipeline suitable for early myopia screening in routine clinical settings by healthcare personnel without specialized informatics training.

The study is structured as follows: Section 2 reviews related work on the use of the YOLO architecture and briefly discusses previous research using YOLOv8. Section 3 outlines the proposed methodology, dataset preparation, and describes the characteristics of YOLOv11. The findings are presented in Section 4. Section 5 discusses our study and provides conclusions.

## 2. Related Work

In [12], the authors tested three CNN architectures (VGG16, VGG19, and InceptionV3) for myopia classification on the Retinal Fundus Multi-Disease Image Dataset (RFMID) [13]. From 495 fundus images, expanded to 2025 through augmentation, models were trained to distinguish normal from myopic eyes. With standard settings (224 × 224 input, 32 batch size, 20 epochs, Adam optimizer), accuracies ranged from 66% to 96% on the original dataset and up to 99.5% after augmentation. Although the strong potential of deep learning for myopia detection was demonstrated, the approach required advanced programming skills, limiting accessibility for non-technical clinicians.

The YOLO (You Only Look Once) family of models represents a widely adopted framework for real-time object detection, known for its speed and competitive accuracy [14,15]. Introduced by [16], YOLO applies a single neural network to the full image, predicting bounding boxes and class probabilities in one evaluation. Over time, several versions of YOLO have been released, each improving on architecture, scalability, and performance trade-offs [17]. The use of the YOLO family ranges from vehicle automation and safety control to surveillance and security, to agricultural applications for plant diseases detection and enhanced fruitlet thinning in commercial orchards, and up to healthcare and medicine, which is our primary area of interest [17,18].

Our previous work with YOLO version 8 and version 9 is extensively documented in [8,11,19,20], where we evaluated these architectures across various optometric and ophthalmologic applications, with particular attention to their accessibility for users without programming expertise. In [11], we explored the segmentation of diabetic retinopathy features—including microaneurysms, hemorrhages, and exudates—using both YOLOv8 and YOLOv9. A custom, manually annotated dataset was developed using Roboflow’s integrated annotation platform [21], which facilitated the labelling process even for non-technical users. We also included optic disc segmentation, given its clinical relevance as a key anatomical landmark of the posterior pole. The best-performing model from YOLOv8 achieved a mean average precision (mAP) of 58.4, with specific average precisions of 98.2% for the optic disc, 50.6% for hemorrhages, and 26.5% for microaneurysms. Notably, when microaneurysms were excluded from the segmentation task, the mAP increased to 77%, highlighting the model’s robustness when focusing on larger or more distinct retinal features. These results demonstrated YOLO’s strong spatial precision and suitability for clinical image analysis, even when used by individuals without prior coding experience. Due to the model’s outstanding performance in localizing the optic disc, we chose to retain and adapt the same detection framework for further research.

Building on these results, in [8], we expanded our investigation by employing YOLOv8 in a novel classification task aimed at diagnosing myopia using full-field fundus photographs. This study marked the first time we applied a YOLO-based detector to perform binary image-level classification, rather than object detection. Despite YOLO’s typical use in detection, the model demonstrated strong generalization capabilities, reaching an accuracy of 84%, recall of 97%, and an F1 score of 89%. These results underscore the model’s potential as a screening tool in clinical settings.

Overall, our findings confirm the versatility of YOLO architectures—especially version 8—not only for precise localization of anatomical structures but also for broader diagnostic applications. Most importantly, the user-friendly design of modern platforms integrating YOLO (such as Roboflow and other no-code tools) makes it possible for clinicians and researchers without a programming background to develop, train, and deploy effective AI-based models. This significantly lowers the barrier to entry for implementing AI in healthcare, especially in resource-limited environments or early-stage research settings.

## 3. Materials and Methods

### 3.1. Dataset

For the present study, we employed the same dataset of 338 retinal fundus images previously collected and described in our earlier publication [8]. The dataset included images from 169 Caucasian patients who attended a single optometry and contact lens practice in Northern Italy for their routine visual examination. Each patient contributed one fundus photograph per eye, resulting in a total of 248 myopic and 90 non-myopic images eligible for analysis. Refractive error was measured under cycloplegia with the supervision of an ophthalmologist. Myopia was defined as a spherical equivalent refraction (SER) ≤ −0.50 D, while non-myopia was defined as SER > −0.50 D. Among the participants, 124 were classified as myopic (74 females) and 45 as non-myopic (27 females). The mean age of the myopic group was 26.74 years (SD = 13.78), whereas the non-myopic group had a mean age of 40.33 years (SD = 17.74). To achieve a satisfactory sample size, all patients in the clinic’s archive who either had a previous diagnosis of myopia or were free from refractive errors were invited to participate via a text message sent to their mobile phones. Recruitment of non-myopic subjects proved particularly challenging, since most patients attending the clinic were myopic. This also reflects the fact that the clinic is highly specialized in myopia management, which partly explains why the mean age of the myopic group was lower than that of the non-myopic group. Only fundus images that met predefined quality standards (adequate focus, illumination, and absence of major artifacts) were considered eligible. Table 1 summarizes the main characteristics of the original dataset and highlights the number of pictures for each class and the mean spherical equivalent refraction (SER) defining the myopic and non-myopic eyes.

Eyes with an SER ≤ −0.50 D were classified as myopic and assigned to the myopic dataset, while those with an SER of > −0.50 D were classified as non-myopic and assigned to the non-myopic dataset [22].

In the current analysis, we adopted a different approach by focusing exclusively on the optic disc region rather than the entire fundus image. To achieve this, we utilized a custom optic disc localization algorithm developed in our prior experimental work [11], based on a YOLOv8 object detection pipeline. This algorithm was used to automatically generate bounding boxes around the optic disc in each image. From these, we cropped the corresponding regions and uniformly resized them to 640 × 640 pixels, creating a new dataset composed solely of optic disc images. The rationale behind this method was to investigate whether image regions centered on the optic disc alone could provide sufficient diagnostic information for downstream classification tasks, such as myopia detection.

The study was conducted in accordance with the principles of the Declaration of Helsinki.

**Figure 1:**
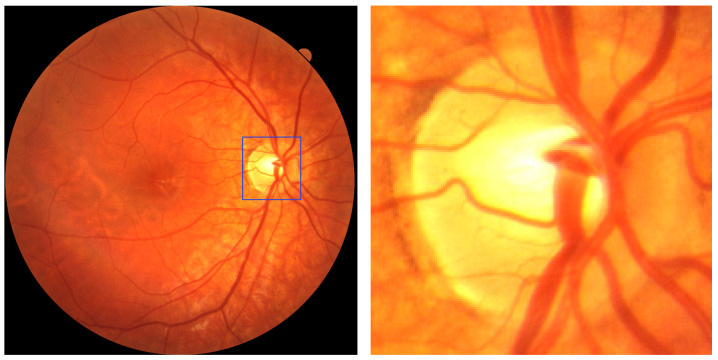
Image processing workflow for optic disc extraction. From (**left**) to (**right**): original retinal fundus image with YOLOv8-generated bounding box (in blue) identifying the optic disc region; cropped and resized optic disc image (640 × 640 pixels) used for model training.

Figure 1 illustrates the image processing pipeline, showing the fundus photo, the YOLO-generated bounding box, and the final cropped and resized optic disc image. All images exhibited sufficient quality in the optic disc region, enabling the successful extraction of the optic disc from each image without exclusions.

### 3.2. Training Parameters

YOLO requires annotated images to perform the classification task. The two annotated datasets were generated and well-structured with the help of Roboflow; then, they were downloaded on the workstation. We split the images into a training set and an internal validation set, ensuring that no image appeared in both sets at the same time. This was possible thanks to Roboflow’s step procedure. We performed image preprocessing steps, including auto-orientation and resizing the images to a resolution of 640 × 640 pixels, which is the recommended size for YOLO. To mitigate the risk of overfitting, we applied various data augmentation techniques, such as horizontal and vertical flips, 90-degree rotations in both clockwise and counterclockwise directions, and flipping the images upside down. These transformations expanded our dataset to 730 images (582 used for training, 98 for internal validation, and 50 for the testing). The batch size, which determines the number of samples processed before updating the model parameters, was set to 4 to conserve computational resources during each epoch. Each YOLOv11 model was trained for 100 epochs, and we implemented an early stopping criterion: if no improvement was observed over ten consecutive epochs, training was halted, as the model was likely to have converged. We then repeated the inference on the test dataset, with 50 completely new images. The experiment was conducted on a system with an Intel Core i7 processor, 64 GB of RAM, and an 8 GB NVIDIA 3070Ti graphics card, comprising the same settings we used in our previous experiments [8,11].

After the YOLOv11 training phase and testing were completed, the five versions of its predecessor, YOLOv8, were trained and then evaluated under identical conditions, using the same training and testing dataset and the same workstation, to ensure a controlled and fair comparison. The two YOLO family versions were then compared to each other, with significant differences in classification performances being analyzed.

### 3.3. You Only Look Once Version 11

In this paper, we used YOLOv8 and YOLOv11, two state-of-the-art CNN models for object detection, segmentation, and classification, accessible online and implemented in PyTorch (version 2.6.0) for segmentation, and real-time detection and classification tasks.

A well-structured and exhaustive description of YOLOv8 can be found in [17,23,24,25] and in our previous published works [8,11], where we explored its usability for medical image analysis and screening method, respectively, in diabetic retinopathy and myopia. In this sub-chapter, we focus on the novelties introduced by YOLOv11.

The new YOLOv11 by Ultralytics represents another advancement in the YOLO family of real-time object detection models, delivering notable gains in detection accuracy, computational efficiency, and architectural flexibility compared to its predecessor, YOLOv8 [26,27]. The difference in inference speed and its competitive performances make YOLOv11 a wise choice, especially in scenarios demanding rapid data processing; it also maintains very good accuracy regarding real-time medical image analysis and evaluation. Table 2 provides an overview of the available YOLOv8 and YOLOv11 variants for project development, detailing the number of used parameters (in millions) and floating-point operations per second (FLOPs), indicating the computational requirements for an image input size of 640 × 640 pixels [28,29].

One of the core architectural innovations of YOLOv11 is the integration of a hybrid transformer–convolutional backbone, which enhances the model’s ability to encode long-range dependencies and global spatial relationships, without substantially compromising inference latency. YOLOv11 further refines the object detection process by focusing on enhanced feature extraction and robust detection capabilities. It introduces the C3k2 (i.e., Cross Stage Partial with kernel size 2) block, a replacement for the previously used C2f block, which significantly improves gradient flow and computational efficiency. Moreover, it utilizes the C2PSA (i.e., Convolutional block with Parallel Spatial Attention) module for improved spatial attention, enhancing feature extraction capabilities, particularly in scenarios involving occlusion and complex backgrounds [17].

**Figure 2:**
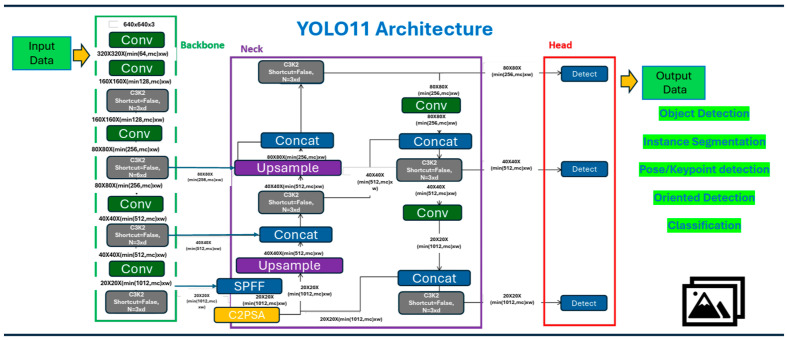
YOLOv11 architecture highlighting the backbone, the neck, and the head between input and output data [18].

These architectural refinements result in superior performance in mean average precision (mAP) across a range of standard benchmarks, particularly under challenging conditions, such as dense object layouts or small-scale targets, with only marginal increases in computational complexity. The architecture, highlighting these critical components, is detailed in Figure 2.

YOLOv11 series achieves an extraordinarily low inference speed, making it the optimal choice for environments where both high speed and precision are critical for efficient automation and real-time decision making, such as in the medical field.

In healthcare, YOLO has been instrumental in assisting and improving diagnostic processes and treatment outcomes. The applications include, but are not limited to, breast and esophageal cancer detection [30,31], skin lesion segmentation [32], brain tumors in MRI images [33] (which showcase the model’s ability to adapt to different needs), and essential tasks.

Although You Only Look Once has been used in multiple computer vision projects across diverse fields, spanning various available versions and even modifying some of them [17], it is still seldom used in optometry and ophthalmology, except for a few rare cases. A PubMed search using the keywords “YOLOv11” AND “glaucoma” or “maculopathy” or “retinopathy” or “myopia” did not reveal any published scientific work. Therefore, the present article represents one of the first applications of this CNN in myopia management and screening process, and in optometry and ophthalmology fields.

### 3.4. Performance Metrics

The performance metrics used to analyze the results of our research outcomes include the following [15]:

- Accuracy (ACC) is defined as the ratio of correct predictions to the total predictions made, as illustrated in Equation (1).
- Precision (P) and recall (R) assess the ratio of true positives in all positive predictions and determine the ratio of true positives in all actual positives, respectively. R measures the model’s effectiveness in identifying all instances of a class. P and R are computed using Equations (2) and (3), respectively. Recall and sensitivity (Se) are calculated using Equation (3).
- Specificity (Sp) calculates the ratio of true negatives in all negative predictions, as shown in Equation (4).
- The F1 score represents the harmonic mean of P and R, providing a balanced evaluation of the model’s accuracy by taking into account false positives and negatives, as demonstrated in Equation (5).
- The receiver operating characteristic (ROC) curve is a graphical representation of a classification model’s performance across all classification thresholds. It shows the trade-off between the true positive rate (TPR) and the false positive rate (FPR). TPR is also known as recall or sensitivity (Equation (3)). The FPR is the ratio of incorrectly identified negative instances to the total actual negative instances, illustrated in Equation (6). As the classification changes, both the TPR and FPR change, and plotting them forms the ROC curve.

(1) $$Accuracy=\frac{TP+TN}{TP+TN+FP+FN}$$

(2) $$Precision=\frac{TP}{TP+FP}$$

(3) $$\mathrm{Recall}\;\mathrm{or}\;\mathrm{Sensitivity}\;\mathrm{or}\;\mathrm{TPR}=\frac{TP}{TP+FN}$$

(4) $$Specificity=\frac{TN}{TN+FP}$$

(5) $$F1\;\mathrm{score}=\frac{TP}{TP+\frac{1}{2}(FP+FN)}$$

(6) $$FPR=\frac{FP}{FP+TN}$$

where TP is true positive, TN is true negative, FP is false positive, and FN is false negative. Precision and recall are valuable metrics for evaluating a model’s performance when class distributions are imbalanced. Specific tasks, such as in medical image analysis, prioritize a higher recall over precision to reduce the rate of false negatives. Therefore, achieving a high recall or sensitivity is prioritized over high precision, as a false negative could lead to an incorrect medical diagnosis and pose risks to a patient’s health [15].

The AUC provides a single numerical summary of the model’s ability to correctly place an object in the corresponding class. When the AUC is close to 1.0, this represents perfect classification, while a value of 0.5 suggests that the model has no discrimination ability (equivalent to a random classifier). Higher AUC values, between 0.8 and 1.0, typically indicate very good model performance.

In our experiment, we choose the best-performing model based on the shortest computational time when two or more models exhibited similar performance.

### 3.5. Statistical Analysis

We conducted the statistical analysis using Microsoft Excel and Jupyter Lab (version 4.2.5). Microsoft Excel was used to calculate the performance metrics based on the TP, TN, FP, and FN values from each confusion matrix. Jupyter Lab was used to compute and plot the ROC curve and AUC on the test dataset. It supports multiple programming languages, especially Python (version 3.12.7), and is popular because it combines code execution, data visualization, and documentation in a single environment. Since we had only one performance measure per model, we applied the bootstrap method to calculate the 95% confidence intervals (CIs). This method does not assume a normal distribution and is therefore very robust.

The Matthews Correlation Coefficient (MCC) and the Negative Predictive Value (NPV) were used to assess the quality of predictions and the likelihood to be truly healthy (in our case, not myopia) if the result is negative or not-myopic, respectively [34,35]. The MCC is a metric used in binary classification and is particularly useful when dealing with imbalanced datasets, where one class (such as myopia) has more examples than the other (such as normal or not myopia). It ranges from −1 to +1, with +1 indicating perfect agreement between predicted and actual values, 0 indicating random guessing, and −1 total disagreement. The formula for the MCC is shown in Equation (7). The NPV is a metric used to answer the following question: “if I have a negative test, what is the probability that I actually do not have the disease?”. In other words, this means that the higher the NPV, the higher and more reliable the test is at ruling out the disease. The definition of the NPV is described by the formula in Equation (8).

(7) $$MCC=\frac{\left(TP\times TN\right)-(FP\times FN)}{\sqrt{\left(TP+FP)(TP+FN)(TN+FP)(TN+FN\right)}}$$

(8) $$NPV=\frac{TN}{TN+FN}$$

Finally, we also performed the bootstrap technique and the DeLong test to compare AUCs and identify the best-performing model. All pairwise comparisons were adjusted for multiple comparisons.

## 4. Results

### 4.1. Internal Validation Outcomes

**Figure 3:**
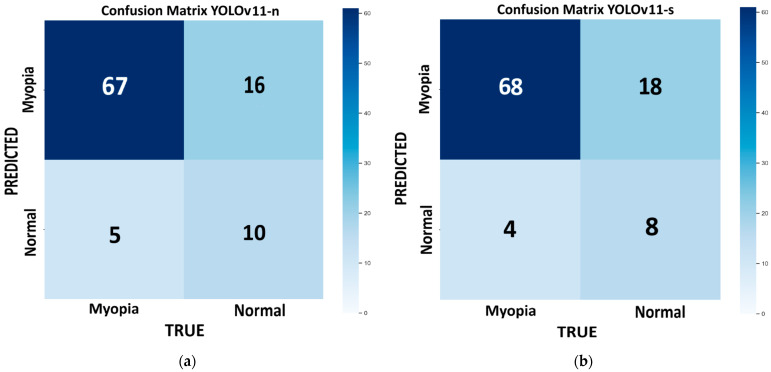

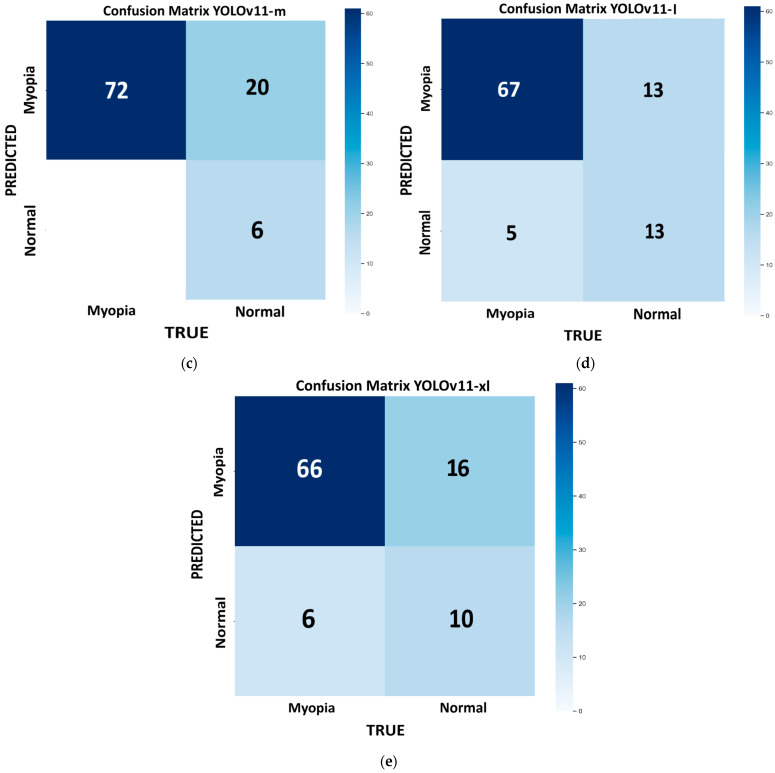
Confusion matrix from each variant of YOLOv11 tested on internal validation set, with number of TP, TN, FP, and FN: (**a**) nano; (**b**) small; (**c**) medium; (**d**) large; and (**e**) extra-large models.

Figure 3 shows the confusion matrix for each model of YOLOv11 tested on the internal validation dataset: nano (Figure 3a), small (Figure 3b), medium (Figure 3c), large (Figure 3d), and extra large (Figure 3e). This dataset consists of 98 images, with 72 for myopic eyes and 26 for healthy eyes. Since we had only one value per model, we used the bootstrap method, which revealed no statistically significant differences in the performance metrics across the different models, except for the m model, which provides the highest recall (100%) but has low precision (78.4%) and very low specificity (23%). With this model, the number of false positives is the highest (FP = 20), and it is not suitable for efficient screening purposes. In medicine, recall is crucial for minimizing false negatives and preventing misdiagnosis. The best-performing model, which combines higher recall, accuracy, and F1 score, with lower computational time, is the large variant (R = 93%; ACC = 82%; F1 score = 88%).

**Figure 4:**
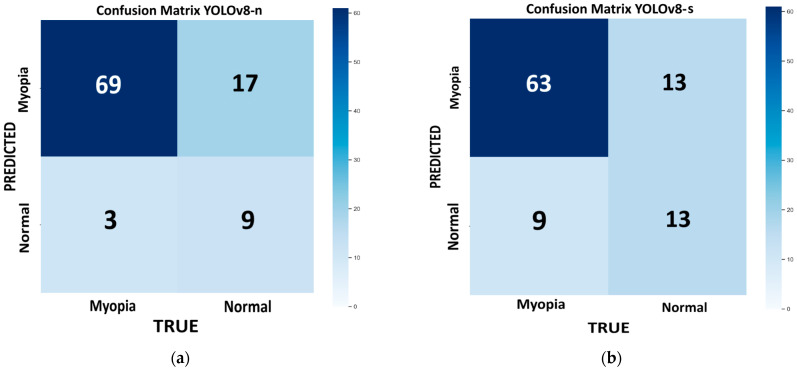

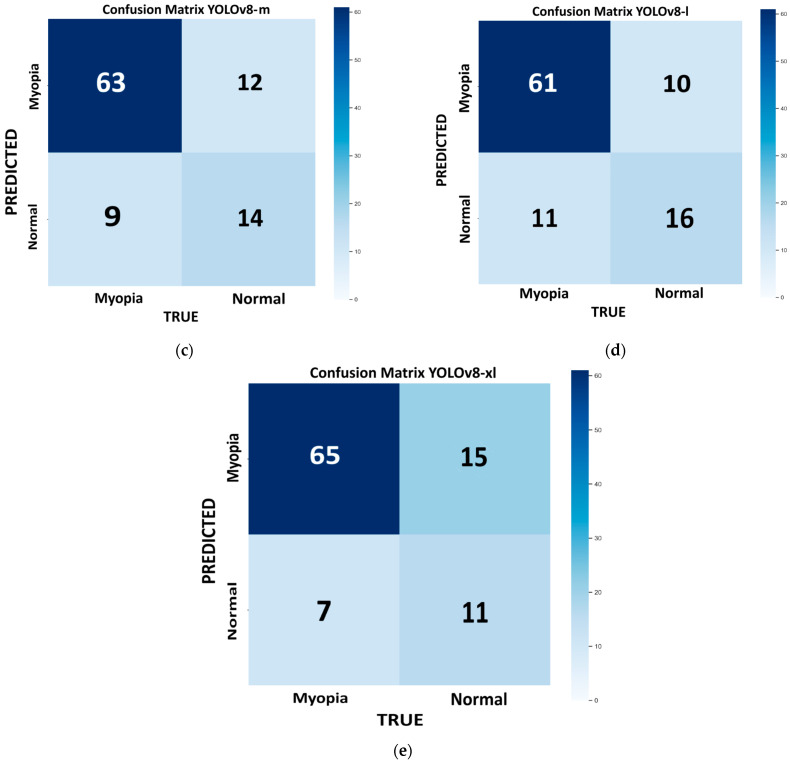
Confusion matrix from each variant of YOLOv8 tested on internal validation set, with number of TP, TN, FP, and FN: (**a**) nano; (**b**) small; (**c**) medium; (**d**) large; and (**e**) extra-large models.

Figure 4 shows the confusion matrix for each model of YOLOv8 tested on the internal validation dataset: nano (Figure 4a), small (Figure 4b), medium (Figure 4c), large (Figure 4d), and extra large (Figure 4e). The bootstrap method revealed no statistically significant differences in the performance metrics across the different models. The best-performing model, which combines higher recall, accuracy, and F1 score with a lower computational time, is the n variant (96%, 80%, and 87%, respectively). However, this model has the highest number of false positives (FP = 17), which can interfere with the proper screening process flow. If we consider a more balanced approach (F1 score = 86%), with a reduced number of false positives (FP = 12), keeping a high recall and accuracy (R = 88% and ACC = 79%), and with reasonable low computational time, the m model provides a good compromise. Table 3 reports the performance metric results for YOLOv8 and v11 variants.

The best-performing models from the YOLOv11 and YOLOv8 families on the internal validation set were YOLOv11-l and YOLOv8-m, respectively. Both offered high recall, accuracy, and F1 score, while maintaining fast inference times. As shown in Table 4, YOLOv11-l outperformed YOLOv8-m in terms of accuracy (82% vs. 79%), recall (93% vs. 88%), and F1 score (88% vs. 86%). Although YOLOv8-m exhibited slightly higher precision (84% vs. 83.8%), YOLOv11-l demonstrated a superior ability to detect true positives, as reflected in its higher MCC (0.542 vs. 0.468). This suggests that YOLOv11-l is better suited for applications where sensitivity is critical, such as in medical diagnoses. Supporting this, the NPV of YOLOv11-l was also higher (0.722 vs. 0.609), indicating greater reliability in ruling out the presence of pathology in negative cases. Overall, the results highlight the robustness of YOLOv11-l, which combines higher sensitivity with a more balanced error profile, making it a strong candidate in settings with class imbalances or where false negatives carry higher clinical risks.

### 4.2. Testing Dataset Results

Table 5 summarizes the performance metrics of the different YOLOv8 and v11 models achieved on the external testing dataset, which consists of 50 images, with 35 images from myopic eyes and 15 from healthy eyes. Among all variants evaluated on the test set, the YOLOv11-l model achieved the best overall performance across all metrics. Specifically, it obtained the highest accuracy (92%), a perfect recall (100%), and the best F1 score (95%), indicating an excellent balance between sensitivity and precision (90%). Notably, it also achieved the highest specificity (73%) alongside YOLOv11-xl, suggesting a reduced rate of false positives and thus a more reliable model in distinguishing negative cases. The YOLOv11-xl model performed similarly well, with slightly lower recall (97%) and F1 score (93%), reflecting a minor compromise in sensitivity but maintaining strong precision (90%) and specificity. The smaller models, YOLOv11-n and YOLOv11-s, also demonstrated high recall (97% and 100%, respectively), confirming their ability to detect positive cases effectively. However, their lower specificity (60% for YOLOv11-n and 53% for YOLOv11-s) and moderate precision (85% and 83%, respectively) suggest a tendency toward false positives, which may limit their suitability in applications where diagnostic certainty is required. The YOLOv11-m variant showed the weakest performance, with the lowest specificity (27%) and accuracy (78%), despite achieving perfect recall. This imbalance indicates that YOLOv11-m is prone to over predicting the positive class, leading to a high rate of false alarms. Overall, YOLOv11-l emerges as the most balanced and robust model, especially in settings where both high sensitivity and high specificity are critical—such as in medical image diagnosis—providing confident positive detections while minimizing false positives.

While the YOLOv11-large clearly outperforms the other models tested, we compared it with the variant that we consider to be the second-best overall—YOLOv11-nano—based on its performance in terms of accuracy, sensitivity, and F1 score, as well as its faster inference speed.

First of all, a direct bootstrap comparison between the nano and small models revealed no statistically significant differences across the performance metrics (*p* > 0.05), so the MCC and NPV were calculated to choose the best between them.

While YOLOv11-small achieved higher values in both NPV (1.00 vs. 0.90) and MCC (0.667 vs. 0.655) compared to YOLOv11-nano, bootstrap analysis showed that these differences were not statistically significant (*p* = 0.4846). These results suggest that, despite small numerical advantages, the two models perform comparably within the limits of the current dataset. Thus, we decided to compare the large variant of YOLOv11 with the nano model.

YOLOv11-large demonstrated significantly better performance than YOLOv11-nano in terms of both MCC (0.811 vs. 0.450; *p* = 0.0445) and accuracy (0.920 vs. 0.860; *p* = 0.0436). The NPV of YOLOv11-large was 100% compared to 90% for YOLOv11-nano, with a mean difference of 0.098, indicating a numerical improvement that was not statistically significant. Overall, these results suggest that YOLOv11-large provides a statistically significant performance gain over YOLOv11-nano in terms of classification consistency and accuracy, while the advantage in NPV, although numerically evident, does not reach statistical significance given the current dataset size. The large model of YOLOv11is still our best choice at this stage of the testing phase.

**Figure 5:**
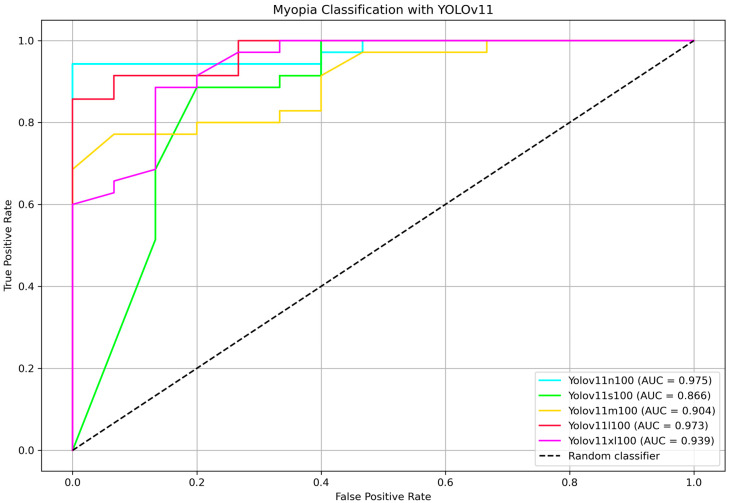
ROC and AUC for YOLOv11 model n, s, m, l and xl calculated on testing dataset. Calculated AUC values are displayed in the bottom right corner.

To further identify the most efficient model for distinguishing between myopia and non-myopia from the optic disc picture, ROC curves and corresponding AUC values were computed for all YOLOv11 variants. Figure 5 shows the ROC curve comparison highlighting the AUC values. Bootstrap-based pairwise comparisons of AUC between YOLOv11 variants revealed no statistically significant differences (all *p* > 0.05). Although some comparisons showed marginal trends (e.g., nano vs. medium, *p* = 0.080), the results suggest that the discriminative performance of these models, as measured by AUC, is statistically comparable within the external testing set. The two highest AUC belongs to the nano and the large models, 97.5% and 97.3%, respectively, showing that the two classifier are comparable (*p* = 0.962). The DeLong test for nano vs. large variants confirmed that they are not statistically different (*p* = 0.990).

**Figure 6:**
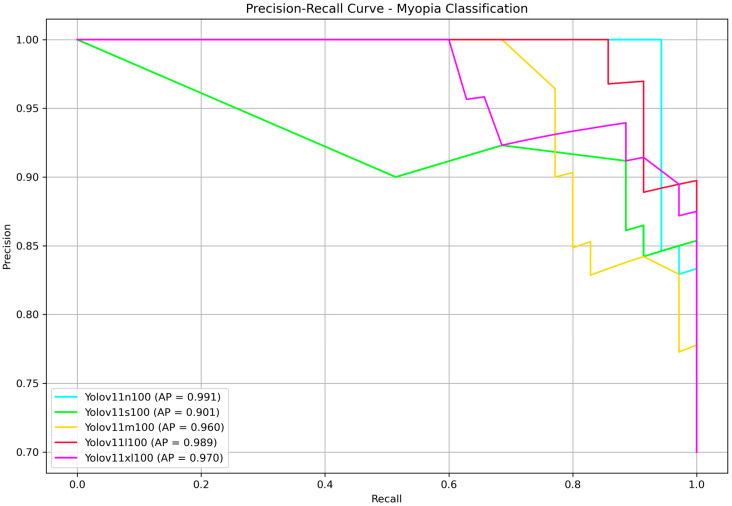
Precision–recall curves for YOLOv11 models n, s, m, l and xl. Calculated average precisions (APs) are displayed in the box in bottom left corner.

Finally, to present our results in a format familiar to the YOLO community, we reported the precision–recall curves along with the corresponding average precision (AP) values in Figure 6. AP, defined as the area under the precision–recall curve, is a widely used metric for evaluating classification performance, particularly in imbalanced datasets. In our binary classification task—discriminating between myopia and normal cases—AP effectively captures the model’s ability to rank predictions across all confidence thresholds. Since only a single positive class (myopia) is considered, AP serves as an appropriate and equivalent metric to the mean average precision (mAP), which is commonly used in multi-class or object detection contexts. No statistically significant differences were found, all the models achieved very high AP. The nano and large models provided once more the highest score, with AP of 99.1% and 98.9%, respectively. Since YOLOv11 is faster than its predecessors, this time we decided to choose the large model as the best performing model at the end of the testing phase.

The YOLOv8-l model achieved the highest overall accuracy (98.0%), precision (97%), recall (100%), specificity (93%), and F1 score (99%). The medium and extra-large variants showed comparable accuracy (92%) and perfect recall (100%), but their specificity was notably lower (73%). Smaller models, such as YOLOv8-n and YOLOv8-s, demonstrated lower accuracy and specificity, with the n model exhibiting particularly low specificity (33%) despite perfect recall. These findings suggest that increasing the model size generally improves diagnostic specificity and overall balanced performance, with YOLOv8-l offering the best trade-off among the tested variants.

Although the large model achieved superior metrics, the difference in performance compared to YOLOv8 medium was not statistically significant (*p* > 0.05). Therefore, at this stage, we selected the medium variant for its reduced computational time and its optimal balance between high accuracy, perfect recall, and solid F1 score, combined with greater efficiency.

Both YOLOv8-m and YOLOv8-l achieved a perfect NPV of 100%, indicating that no positive cases were misclassified as negative and confirming their strong reliability in safely excluding the pathological condition. However, when comparing the overall quality of predictions using the MCC, YOLOv8-l significantly outperformed YOLOv8-m (0.953 vs. 0.811). A bootstrap test confirmed that this difference was statistically significant (*p* = 0.0473), highlighting that the large variant offers a more robust and balanced classification performance across all elements of the confusion matrix.

In the medical context, sensitivity (recall) is critical to minimizing false negatives and avoiding misdiagnoses. Based on this, the YOLOv8-l variant is the best-performing model if longer inference times are acceptable. Alternatively, the medium variant provides faster classification with only a slight trade-off in performance while maintaining a high diagnostic ability.

**Figure 7:**
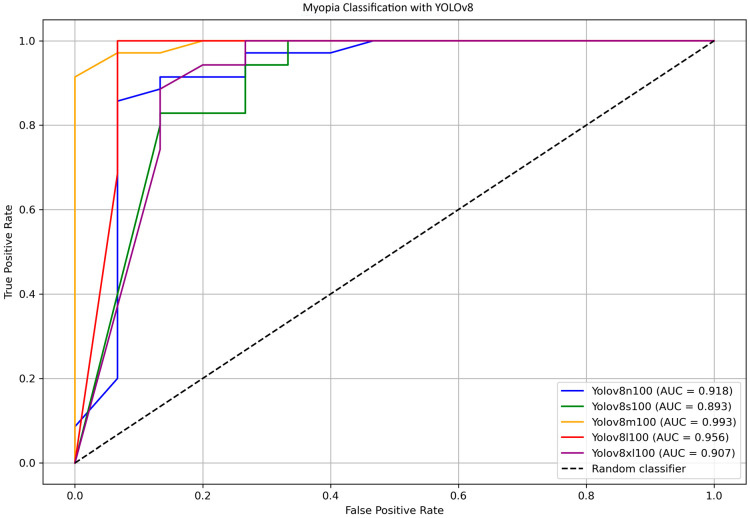
ROC and AUC for YOLOv8 models n, s, m, l and xl calculated on test dataset. Calculated AUC values are displayed in the bottom right corner.

To further identify the most efficient model for distinguishing between myopia and non-myopia, ROC curves and corresponding AUC values were computed for all YOLOv8 variants. Figure 7 shows the ROC curve comparison for YOLOv8 models n, s, m, l, and xl, highlighting the AUC values. Bootstrap analysis was employed to estimate confidence intervals for the AUCs and to assess the overall differences among the various models. The results indicate that the difference in the AUC between YOLOv8-s and YOLO8-m is statistically significant (*p* = 0.018); differences between other pairs of models were not significant. The direct comparison between YOLOv8-m (AUC = 0.993) and YOLOv8-s (AUC = 0.893) was further investigated using the DeLong test, which found no statistically significant differences (*p* = 0.398).

The medium variant achieved an AUC of 0.993, while the large variant showed a slightly lower AUC of 0.956. The DeLong test, which was used for comparing these two models, confirmed no statistically significant difference in the AUC (*p* = 0.762), supporting the notion that both models provide a similarly excellent discriminatory power between myopia and non-myopia.

Overall, these findings confirm that the YOLOv8 models, particularly the medium and large variants, deliver a high diagnostic accuracy with excellent sensitivity and specificity. The medium model offers a compelling balance of performance and computational efficiency, making it suitable for practical applications where inference time and computational power are a concern; on the other hand, the large model maximizes all the performances if longer processing time is acceptable.

**Figure 8:**
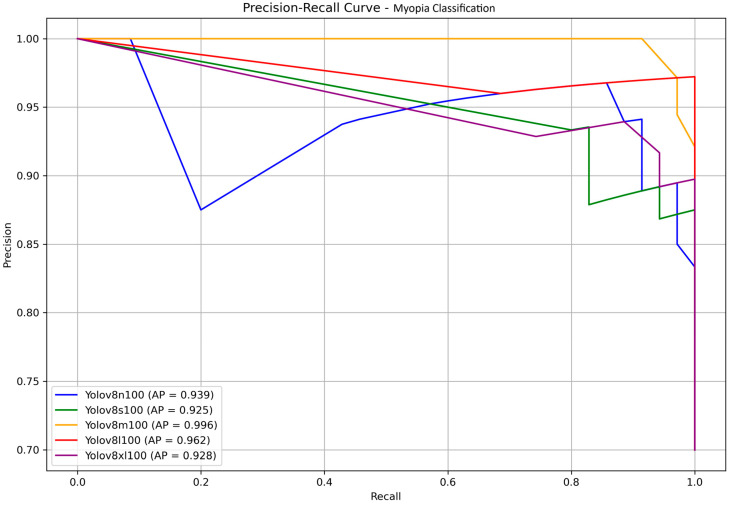
Precision–recall curves forYOLOv8 models n, s, m, l, and xl. Calculated average precisions (APs) are displayed in the box in bottom left corner.

The precision–recall curves of the YOLOv8 models, along with their corresponding AP values, are presented in Figure 8 for a clear comparison. The medium and large models once again achieve the highest scores, with AP values of 0.996 and 0.962, respectively, showing no statistically significant differences. Considering all the above observations, and assuming a scenario without computer science expertise and with limited resources, the medium model emerges as the most suitable choice for the final testing stage.

### 4.3. Summary of the Results

Among the evaluated models, YOLOv11-l and YOLOv8-m emerged as the top performers on the test dataset. Both models achieved identical confusion matrices, with 35 TP, 11 TN, 4 FP, and 0 FN, resulting in perfect sensitivity (100%); high precision (90%), accuracy (92%), and specificity (73%); and a balanced F1 score of 95%. These metrics (as fully shown in Table 6), suggest that the models have an excellent ability to detect myopia, which is particularly important in clinical settings. Despite the equivalence in basic classification performance, YOLOv8-m showed slightly higher values in terms of the area under the ROC curve (AUC = 99.3% vs. 97.3%—*p* > 0.05) and average precision (AP = 99.6% vs. 98.9%—*p* > 0.05), suggesting a marginally superior confidence calibration and ranking ability across thresholds. This indicates that, while both models are equally effective at binary classification, YOLOv8-m may offer improved robustness when confidence scores are critical, such as in decision support systems or threshold tuning scenarios. Both YOLOv11-l and YOLOv8-m models achieved an MCC of 81%, indicating a strong positive correlation between the predicted and actual class labels. This value reflects a robust global performance, as the MCC incorporates all elements of the confusion matrix (TP, TN, FP, FN) into a single metric. Unlike accuracy, which can be misleading in the presence of class imbalances, the MCC remains a reliable measure of overall predictive quality. An MCC above 0.80 is generally interpreted as an excellent result, demonstrating that the models are not only highly sensitive and precise but also balanced in their error distributions. This supports their suitability for diagnostic applications, where both correct identification and reliable exclusion of pathological cases are essential. Therefore, both models are well suited for deployment in diagnostic applications, with YOLOv8-m showing a slight edge in probabilistic output evaluations.

When including the second-best variants, YOLOv8-l notably achieved superior overall classification with an MCC of 95%, no false negatives, and a specificity of 93%, indicating high robustness in correctly identifying both positive and negative cases. In contrast, YOLOv11-n, while maintaining high recall (97%), was penalized by a higher number of false positives (FP = 6), resulting in a lower MCC (66%). These results highlight the importance of balancing sensitivity and specificity and emphasize the robustness of YOLOv8-l in diagnostic scenarios where overall predictive reliability is critical.

Both YOLOv8 and YOLOv11 architectures demonstrated high and clinically meaningful performance in the automated detection of myopia through optic disc photographs. The results suggest that these models are suitable for deployment in diagnostic pipelines aimed at supporting eye care practitioners, without coding expertise, in early screening and assessment tasks. Within the YOLOv11 family, the large variant emerged as the most appropriate choice, balancing high recall, precision, and robustness. For YOLOv8, both the medium and large variants showed excellent performance, with YOLOv8-l standing out as the most accurate and reliable model overall. These findings confirm that both model families are viable options for the AI-assisted diagnosis of myopia and underline the potential of lightweight object detection networks in medical imaging applications.

### 4.4. Activation Maps Visualization

**Figure 9:**
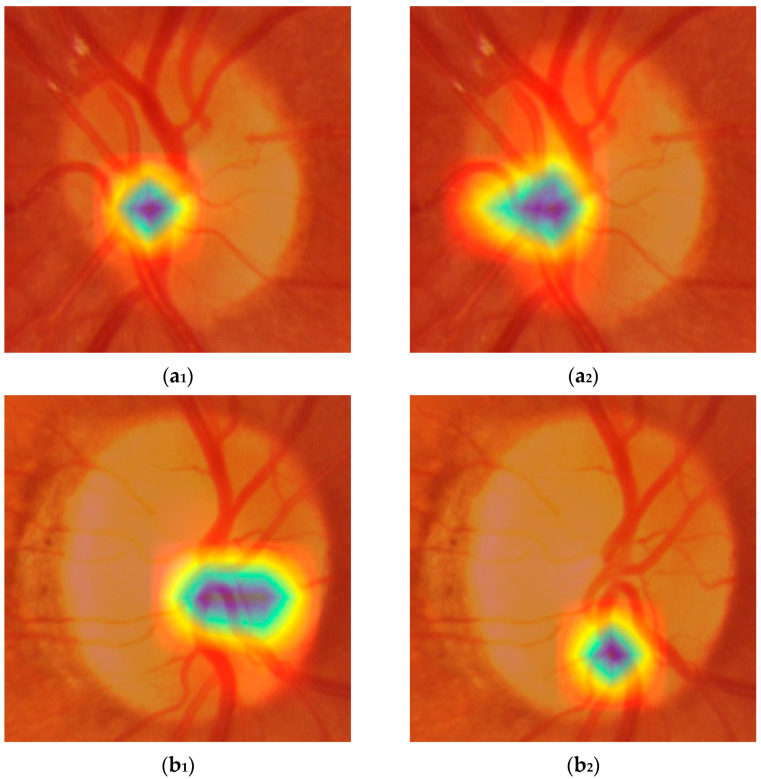

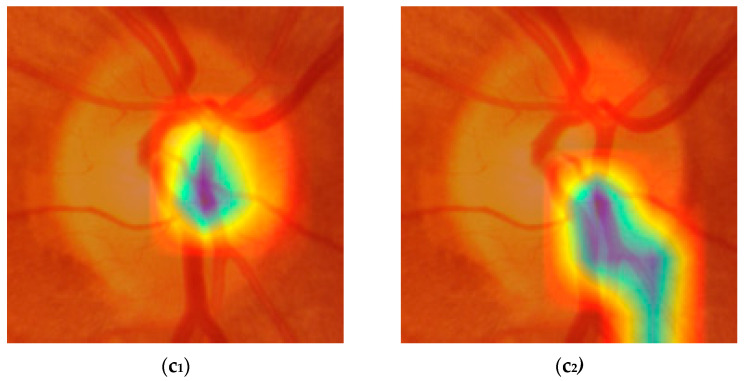
Activation maps generated with the Grad-CAM technique for YOLOv8-m (**a_1_**,**b_1_**,**c_1_**) and for YOLOv11-l (**a_2_**,**b_2_**,**c_2_**) for the same OD pictures: (**a**) example of correct prediction; (**b**) example of incorrect prediction; and (**c**) example of correct vs. incorrect prediction. Parula color map was used, where red color means maximum and blue color means minimum activation.

Activation maps were generated and used to better understand how the models made their predictions. We used the Grad-CAM technique (Gradient-weighted Class Activation Mapping) [36], which enables a visualization of the image regions that most strongly influence the model’s classification, and the Parula color map, allowing for a more precise and balanced visual representation of activation intensities. The Parula color map enhances the clinical interpretability of maps compared to traditional maps, such as Jet. This methodological combination provided a reliable visual assessment of the differences in attention patterns between the YOLOv8-m and YOLOv11-l models. Figure 9 shows six examples of activation maps (three from YOLOv8-m and three from YOLOv11-l), based on the following: (a) both models correctly predicting the actual ground truth; (b) both models making a wrong prediction; and (c) one model predicting the outcome correctly and the other not.

Figure 9(a_1_,a_2_) show how the models correctly predict the absence of myopia. Although both models focused on the same portion of the optic disc, YOLOv8-m showed a more localized and distinct pattern, with activation confined to peripheral regions of the optic disc, leaving the central zone inactive. YOLOv11-l instead involved a larger portion of the same OD region, including the nasal side of the OD margin and part of the superior vascular segments. In both cases, the cup was poorly activated, which is consistent with the plausible absence of myopia.

In Figure 9(b_1_,b_2_), the examined models incorrectly classified the picture as if it was from a myopic eye. The region of activation is focused on the paracentral portion of the OD, with the cup still inactive, with YOLOv8-m being larger and growing more horizontally compared to YOLOv11-l, which is more localized and downward. At this point, it is already difficult to understand what the differences are that let the models predict the ground truth correctly or not. That is because the activated regions represented in the maps are very similar and sometimes confounding. The same could be said for the last example case reported in Figure 9(c_1_,c_2_), where YOLOv8-m correctly classifies a normal eye as a true negative, while YOLOv11-l identifies it as myopic (i.e., a false positive). In Figure 9(c_1_), the pattern is extremely similar to the one shown by (a_1_) and (a_2_), but it is also very hard to state whether it is different from (b_1_) and (b_2_). The activation gradient is well-defined and concentrated, suggesting that the model identifies a discriminating anatomical feature with high confidence; however, we cannot say with certainty whether it depends on the characteristics of the optic disc margin or on localized variations in color and texture. This also makes it difficult to interpret the pattern presented in Figure 9(c_2_) which, despite having a downward smear and a less regular appearance, does not show any obvious signs that distinguish it from the others. This problem makes it difficult to correctly interpret the activation maps both when the predictions are accurate and when they are not. Certainly, further investigation is needed to better understand which characteristics of the optic disc represent the true discriminant for effectively differentiating between myopia and non-myopia.

## 5. Discussion

We successfully created an image dataset consisting of optic disc-centered pictures from myopic and healthy eyes, categorized by eye SER, using our custom-built YOLOv8 segmentation algorithm [14]. This dataset has a strong potential for developing AI-based methods for objective myopia screening based on objective measurable indicators.

Our results suggest that both YOLOv11 and YOLOv8 architectures can classify optic disc images for myopia diagnosis with encouraging performance across accuracy, recall, and F1 score, even in cases of low-to-moderate myopia with an average SER (SD) of −3.30 (3.15) diopters. Nonetheless, these findings should be interpreted cautiously. The differences observed among the model variants and between the two architectures, although present, were relatively small and may partly reflect the limited size and composition of the dataset rather than true performance disparities. Furthermore, given the single-centered and demographically homogeneous nature of the data, it remains uncertain whether these results would hold in broader, more diverse clinical settings.

In the internal validation set, YOLOv11-l offered the best trade-off between recall (93%), accuracy (82%), and F1 score (88%), while maintaining a low inference time and computational cost. In contrast, YOLOv11-m achieved a perfect sensitivity (100%) but very low specificity (23%) and a high number of false positives (FP = 20), making it unsuitable for screening purposes that require a balance between sensitivity and specificity. Within the YOLOv8 family, the nano variant performed well, balancing diagnostic metrics (ACC = 80%, R = 96%, F1 score = 87%) with the processing speed, though at the cost of lower specificity (Sp = 35%) and more false positives (FP = 17). A more balanced compromise was achieved by YOLOv8-m (ACC = 79%, R = 88%, F1 score = 86%, FP = 12), offering good performance with acceptable computational demands.

In the external independent test set, YOLOv11-l stood out with 100% sensitivity, 92% accuracy, an F1 score of 95%, and an MCC of 0.811. Compared to YOLOv11-nano, it had a statistically significant advantage (*p* < 0.05), confirming its robustness. Meanwhile, YOLOv8-l outperformed YOLOv8-m in specificity (93% vs. 73%), with a higher MCC (0.951) and a statistically significant difference (*p* = 0.0473) while a maintaining similar AUC and AP. These findings indicate that both model families are viable for clinical applications: YOLOv11-l is better suited for computationally constrained scenarios, while YOLOv8-l is better suited in scenarios where maximum diagnostic precision is required.

A direct comparison between YOLOv11-l and YOLOv8-m revealed a similar performance in accuracy (92%), precision (90%), recall (100%), specificity (73%), and F1 score (95%). However, YOLOv8-m showed a slight advantage in the AUC (0.993 vs. 0.973) and average precision (AP: 0.996 vs. 0.989), suggesting a better discriminative ability. Conversely, YOLOv11-l was more efficient, with fewer parameters (25.3 M vs. 46.0 M) and FLOPs (86.9 B vs. 220.5 B), making it more suitable for low-power hardware or in real-time applications. These results support the use of both architectures in automated myopia screening based on retinal fundus images, particularly when combined with optic disc localization. Statistical tests (bootstrap methods and DeLong’s test) confirmed that differences in the AUC between the top-performing models were not significant (*p* = 0.375), emphasizing that model choice should depend more on operational constraints—such as hardware availability or the need for real-time inference—than on marginal differences in classification metrics.

YOLOv11 integrates transformer-based components and optimized training strategies, which improve generalization, especially in challenging or imbalanced datasets. Nonetheless, it struggles with small, rotated, or low-resolution objects due to architectural limitations that reduce its representational capacity [17]. Some overfitting was also observed with limited or homogeneous data, highlighting the need for stronger regularization techniques or more robust data augmentation strategies.

YOLOv8, although mature in real-time object detection, remains demanding in terms of computational resources, limiting its applicability on low-power devices or in resource-limited environments [37]. Future work should aim at optimizing both architectures, incorporating context-aware training and adaptive methods to handle heterogeneous inputs and operational conditions.

Beyond model limitations, this study has methodological constraints. First, the external validation was conducted on a relatively small test set (50 images), which may not capture the full variability of real-world clinical practice and therefore limits this study’s generalizability. In addition, the dataset used for model development comprised only 338 retinal images collected from a single clinic, with patients belonging to a relatively homogeneous, non-ethnically diverse population. There was a marked difference in the age distribution between the myopic and non-myopic groups, which could have influenced classification performance. In addition, the study design did not allow for the evaluation of different degrees of myopia; instead, the analysis was restricted to a binary classification (myopia vs. non-myopia).

Together, these factors restrict the generalization of our findings. Larger, multi-center studies with more diverse populations are needed before these AI models can be considered for routine clinical use. Future research should also assess their usability by non-expert personnel and, most importantly, confirm their robustness through stronger external validation.

The analysis was performed on fixed-resolution images (640 × 640), without exploring the impact of different resolutions or image qualities. Additionally, although the confusion matrices were identical, the slight differences observed between YOLOv11-l and YOLOv8-l in the AUC and AP suggest latent differences in score calibration, warranting further investigation.

Furthermore, our analysis focused on standard classification metrics without addressing model interpretability, calibration curves, or decision threshold optimization—factors increasingly relevant in clinical decision support systems. Future research should include these aspects and evaluate performance on external, multi-center, and multi-ethnic datasets to assess generalizability across real-world contexts and diverse clinical settings. In terms of architectural improvements, an important next step would be to repeat the experiment and compare the results using more recent versions of the YOLO algorithm beyond YOLOv8 and YOLOv11. Updated versions, such as YOLOv12 or future iterations, promise improvements in accuracy, efficiency, and generalization ability. These newer models are designed to process images faster and with greater precision, potentially enhancing the detection of subtle features—an essential aspect in clinical diagnostics. Furthermore, they typically incorporate advances in network architectures, optimization strategies, and improved handling of small objects or imbalanced datasets, which could address some of the current limitations observed. As computer vision continues to evolve, staying aligned with the latest developments is crucial for maximizing model performance and ensuring clinical applicability.

Future improvements could also involve evaluating model performance across different age groups, as age-related variations in myopia may affect the classification accuracy. Refining SER thresholds—particularly by including more cases near the −0.50 D cutoff and higher hyperopic values—could help the model better distinguish low myopia from hyperopia. Leveraging transfer learning from larger, diverse datasets may also enhance generalization and improve accuracy across broader populations. Finally, the integration of multimodal data—such as OCT images, visual fields, and clinical parameters—could further improve model performance by capturing complementary diagnostic information.

Furthermore, expanding the model’s detection and classification capabilities to include other fundus-visible eye diseases, such as glaucoma [38,39], would enhance its clinical value and broaden its applicability in ophthalmology. Training the algorithm with optic disc images from glaucomatous and non-glaucomatous patients may offer a smart and cheaper screening method than conventional OCT analysis. The optic disc, being a key retinal landmark, is readily captured and stored in a digital format, and its analysis can be facilitated by portable fundus cameras or smartphone-based devices [40,41]. These tools could benefit from the proposed approach, supporting clinicians with AI-assisted screening and diagnostic capabilities even in resource-limited settings.

In summary, while YOLOv8-l showed the highest overall accuracy, YOLOv11-l was a strong alternative with superior efficiency. Final model selection should therefore be guided by application-specific requirements: YOLOv11-l is ideal where speed and low-resource deployment are critical, whereas YOLOv8-l is preferred when diagnostic precision is the top priority.

In conclusion, this study provides preliminary evidence that YOLOv8 and YOLOv11 models may be able to classify optic disc images for myopia diagnosis, showing a potentially favorable balance between accuracy and efficiency. While YOLOv8-l appeared to perform best overall, YOLOv11-l could represent a promising option in resource-limited settings. However, these observations are based on a relatively small, single-center dataset and should therefore be interpreted with caution. Further research involving larger, more diverse and well-balanced cohorts, as well as thorough evaluations of model calibration, interpretability, and adaptability to real-world clinical workflows, will be essential before considering any practical implementations.

## Figures and Tables

**Table 1:** Summary description of patients’ characteristics and dataset details [8].

Characteristics	Myopic	Non-Myopic	Total
Participants	124 (74 F, 50 M)	45 (27 F, 18 M)	169
Sex (%)	59.7% F/40.3% M	60% F/40% M	59.8% F/40.2% M
Prevalence in Sample	73.4%	26.6%	---
Images (n)	248	90	338
Mean age (SD)	26.74 (13.78)	40.33 (17.74)	30.36 (16.05)
Median age (IQR)	25 (15–34)	44 (28–53)	28–29 (16–44)
Age range (min–max)	9–61	7–84	7–84
Mean SER (SD)	−3.30 (3.15) D	+0.96 (1.31) D	−2.17 (3.36) D
Data collection period	March–August 2023	idem	idem

F: female; SD: standard deviation; IQR: interquartile range; D: diopter; SER: spherical equivalent refraction.

**Table 2:** YOLO version 8 and version 11 model variant parameters for an image input size of 640 × 640.

Model Variant	YOLOv8—Parameters (M)	YOLOv11—Parameters (M)	YOLOv8—FLOPs (B)	YOLOv11—FLOPs (B)
Nano (n)	3.4	2.6	12.6	6.5
Small (s)	11.8	9.4	42.6	21.5
Medium (m)	27.3	20.1	110.2	68.0
Large (l)	46.0	25.3	220.5	86.9
Extra large (xl)	71.8	56.9	344.1	194.9

**Table 3:** The results obtained by the proposed approach with all the available variants of YOLOv8 and YOLOv11 on the internal validation test set.

Model	ACC	P	R	Sp	F1 Score
YOLOv8-n	0.796	0.802	0.958	0.346	0.872
YOLOv8-s	0.776	0.829	0.875	0.500	0.851
YOLOv8-m	0.786	0.840	0.875	0.540	0.856
YOLOv8-l	0.786	0.859	0.847	0.615	0.853
YOLOv8-xl	0.776	0.813	0.903	0.423	0.856
Model	ACC	P	R	Sp	F1 Score
YOLOv11-n	0.786	0.807	0.931	0.385	0.865
YOLOv11-s	0.776	0.790	0.944	0.308	0.861
YOLOv11-m	0.796	0.783	1.000	0.231	0.878
YOLOv11-l	0.817	0.838	0.931	0.500	0.882
YOLOv11-xl	0.776	0.805	0.917	0.385	0.857

**Table 4:** The results obtained by the two best approaches, YOLOv11 large and YOLOv8 medium, on the internal validation test set.

Model	ACC	P	R	Sp	F1 Score	MCC	NPV
YOLOv11-l	0.817	0.838	0.931	0.500	0.882	0.542	0.722
YOLOv8-m	0.786	0.840	0.875	0.540	0.856	0.468	0.609

**Table 5:** The results obtained by the proposed approach with all the available variants of YOLOv8 and YOLOv11 on the testing dataset.

Model	ACC	P	R	Sp	F1 Score
YOLOv8-n	0.800	0.778	1.000	0.333	0.875
YOLOv8-s	0.860	0.868	0.943	0.667	0.904
YOLOv8-m	0.920	0.897	1.000	0.733	0.946
YOLOv8-l	0.980	0.972	1.000	0.933	0.986
YOLOv8-xl	0.920	0.897	1.000	0.733	0.946
Model	ACC	P	R	Sp	F1 Score
YOLOv11-n	0.860	0.850	0.971	0.600	0.907
YOLOv11-s	0.860	0.833	1.000	0.533	0.909
YOLOv11-m	0.780	0.761	1.000	0.267	0.864
YOLOv11-l	0.920	0.897	1.000	0.733	0.946
YOLOv11-xl	0.900	0.895	0.971	0.733	0.931

**Table 6:** The results obtained by the two best approaches after the testing phase: YOLOv11 large and YOLOv8 medium (both in bold). The second-best-performing models for both the YOLO families are shown as well, as they can be considered a good alternative.

Model	ACC	P	R	Sp	F1 Score	AUC	AP	MCC
**YOLOv11-l**	**0.920**	**0.897**	**1.000**	**0.733**	**0.946**	**0.973**	**0.989**	**0.811**
**YOLOv8-m**	**0.920**	**0.897**	**1.000**	**0.733**	**0.946**	**0.993**	**0.996**	**0.811**
YOLOv11-n	0.860	0.850	0.971	0.600	0.907	0.975	0.991	0.655
YOLOv8-l	0.980	0.972	1.000	0.933	0.986	0.953	0.962	0.951

## Data Availability

The optic disc image dataset created for this research can be made available upon request.
